# Bacterial community structure is indicative of chemical inputs in the Upper Mississippi River

**DOI:** 10.3389/fmicb.2014.00524

**Published:** 2014-10-08

**Authors:** Christopher Staley, Trevor J. Gould, Ping Wang, Jane Phillips, James B. Cotner, Michael J. Sadowsky

**Affiliations:** ^1^BioTechnology Institute, University of MinnesotaSt. Paul, MN, USA; ^2^Department of Biology Teaching and Learning, University of MinnesotaSt. Paul, MN, USA; ^3^Department of Ecology, Evolution, and Behavior, University of MinnesotaSt. Paul, MN, USA; ^4^Department of Soil, Water and Climate, University of MinnesotaSt. Paul, MN, USA

**Keywords:** diversity, ecology, environmental, recreational water, metagenomics, water quality

## Abstract

Local and regional associations between bacterial communities and nutrient and chemical concentrations were assessed in the Upper Mississippi River in Minnesota to determine if community structure was associated with discrete types of chemical inputs associated with different land cover. Bacterial communities were characterized by Illumina sequencing of the V6 region of 16S rDNA and compared to >40 chemical and nutrient concentrations. Local bacterial community structure was shaped primarily by associations among bacterial orders. However, order abundances were correlated regionally with nutrient and chemical concentrations, and were also related to major land coverage types. Total organic carbon and total dissolved solids were among the primary abiotic factors associated with local community composition and co-varied with land cover. *Escherichia coli* concentration was poorly related to community composition or nutrient concentrations. Abundances of 14 bacterial orders were related to land coverage type, and seven showed significant differences in abundance (*P* ≤ 0.046) between forested or anthropogenically-impacted sites. This study identifies specific bacterial orders that were associated with chemicals and nutrients derived from specific land cover types and may be useful in assessing water quality. Results of this study reveal the need to investigate community dynamics at both the local and regional scales and to identify shifts in taxonomic community structure that may be useful in determining sources of pollution in the Upper Mississippi River.

## Introduction

The Mississippi River is an important natural resource that is used as a source for drinking water by many cities, as well as for recreational, agricultural, and industrial purposes. The Upper Mississippi River Basin (UMRB), however, has a well-documented history of contamination by heavy metals, including mercury and lead that peaked in the 1960s (Balogh et al., 1999, 2009; Wiener and Sandheinrich, 2010). The UMRB is also impacted by chemicals, including the pesticide DDT, herbicides, and polychlorinated biphenyls (Pereira and Hostettler, 1993; Wiener and Sandheinrich, 2010). In addition, nutrient loading, especially nitrogen and phosphorus, primarily from agricultural runoff (Schilling et al., 2010), has been a persistent concern, with estimates that the UMRB contributed as much as 43 and 26% of total nitrogen and phosphorus, respectively, to the northern Gulf of Mexico from 2001 to 2005 (United States Environmental Protection Agency, 2007). Furthermore, effluent from urban and suburban wastewater treatment plants has been shown to increase organic and inorganic nutrient content downstream in temperate river systems and to decrease bacterial diversity (Drury et al., 2013). While the input of many pollutants to the UMRB has declined since passage of the Clean Water Act and other regulatory measures in the 1970s (Balogh et al., 2009; Wiener and Sandheinrich, 2010), non-point sources of pollution remain a contemporary concern (Wiener and Sandheinrich, 2010).

In addition to long-recognized nutrient contamination of the UMRB, several other pollutants, including fecal contamination, traditionally measured by using fecal indicator bacteria (*Escherichia coli* and *Enterococcus*) (United States Environmental Protection Agency, 2002, 2012), antimicrobials, other pharmaceuticals, and personal care product pollutants have potential negative impacts on the river's water quality. Recently, reaches of the Mississippi River between the Coon Rapids Dam and Minneapolis, as well as a section near Saint Paul, MN have been deemed impaired due to elevated concentrations of *E. coli* (Russell and Weller, 2012). Furthermore, pharmaceuticals such as acetaminophen and caffeine have also been detected in the Mississippi River and are likely contributed from wastewater discharge and runoff (Ellis, 2006; Zhang et al., 2007). While elevated nutrient and bacterial concentrations, and the presence of potentially toxic xenobiotic compounds, suggest negative impacts on ecosystem processes, they provide little indication as to the source(s) of contamination, and little is known about resultant effects on bacterial community structure and ecosystem functioning as a result of their presence in the UMRB.

Many recent studies evaluating variation in microbial community structure in response to environmental gradients have relied on indirect measures and the use of statistical methods, such as non-metric multidimensional scaling, Mantel tests, and Spearman rank correlations, among others (Fortunato et al., 2012; Portillo et al., 2012; Brandsma et al., 2013; Staley et al., 2013). While these methods provide valuable information, such as how bacterial communities vary in response to changes in salinity and depth on a regional scale (Fortunato et al., 2012), local and temporal differences in microbial assemblages can be difficult to determine due to considerable data reduction using these methods such that an entire study period or study area is typically analyzed and relationships on a local scale may be overlooked. Furthermore, spatial alteration in community structure is difficult to disentangle from changes due to environmental gradients, especially when multiple sources of variation co-vary (Fortunato and Crump, 2011; Fortunato et al., 2012). Local similarity analysis (LSA) has been proposed as a method to explain complex, non-linear relationships between microbial assemblages and co-varying environmental parameters on a local scale that can be displayed in a graphical format (Ruan et al., 2006). Extended LSA (eLSA) is a recent expansion of this analysis to incorporate replicate sample data (Xia et al., 2011), and this method has been subsequently used to characterize seasonal relationships within and between microbial taxa and environmental factors in the English Channel (Gilbert et al., 2012).

In addition to being able to better evaluate complex inter- and intra-community responses to environmental variables in high-throughput DNA sequencing datasets, studies of river water have suggested that the microbial communities may change predictably in response to specific types of anthropogenic inputs or contaminant sources (Unno et al., 2010; Staley et al., 2013). Recent development of several analytical software packages has allowed for relatively rapid assignment of operational taxonomic units (OTUs) to specific sources (Knights et al., 2011; Unno et al., 2012), and these have been used in the study of surface water to identify human-specific fecal contamination (Unno et al., 2012; Newton et al., 2013). These studies suggest that evaluation of the entire bacterial community composition may be a useful water quality monitoring tool, potentially providing accurate assessment of the magnitude and distribution of contamination from a variety of sources. In addition to inputs of specific non-indigenous taxa from allochthonous sources, shifts in bacterial community structure may also be associated with the introduction or increase in concentration of specific chemical contaminants, including pharmaceuticals or heavy metals.

In the current study, we characterized concentrations of nutrients and xenobiotic compounds present in the water column of the UMRB in Minnesota during the summers of 2011 and 2012 in order to determine how chemical inputs to the river influenced bacterial community structure. We hypothesized that associations of bacterial orders with nutrient and chemical concentrations would vary both locally and regionally in response to different sources of contamination. To evaluate local associations, all environmental parameters were modeled into an association network with abundances of bacterial orders, and a predictive Bayesian network was developed to display significant, directional associations over a regional scale. Regional bacterial community structure was further hypothesized to be related to specific land-coverage types that are likely to be influencing chemical gradients throughout the river. While previous studies have identified specific taxa associated with human fecal contamination and wastewater discharge (Drury et al., 2013; Newton et al., 2013), the wide variety of analytes measured here, as well as the large study area, offered a more thorough evaluation of how land-use practices and nutrient concentrations might co-vary, and how bacterial communities vary in response to anthropogenically-impacted chemical contributions both locally and regionally. Furthermore, the ability to associate specific bacterial taxa with discrete types of contamination will prove useful in future water quality monitoring, the adoption of best management practices, and development of total maximum daily load (TMDLs).

## Materials and methods

### Sampling collection and processing

Samples were collected during early summer (May through July) in 2011 and 2012 from 11 sites along the Upper Mississippi River and major contributing rivers near their confluences with the Mississippi River as previously described (Staley et al., 2013). Locations of sampling sites are listed beginning at the headwaters near Lake Itasca to the southern border of Minnesota (near La Crescent; Table 1). Water samples (40 L) were collected in sterile 20 L carboys, transported to the lab, and either processed immediately or stored at 15°C overnight and processed the following day (Staley et al., 2013). Briefly, samples were strained through sterile cheesecloth and sequentially filtered (5–10 μm) to remove debris and aggregate bacteria. A subset of planktonic bacterial cells were subsequently captured on a 0.45-μm-pore-sized polyethane sulfonate filters. The effects of filtration and filter pore-size on bacterial community characterization have been previously explored in our laboratory (Staley et al., 2013), and the 0.45-μm pore size was selected to allow the most efficient filtration of large volumes of water. However, while the 0.45 μm pore size allows efficient filtration of large volumes of water, only larger, free-living planktonic bacteria can be characterized by this method. Cells were elutriated by vortexing filters in pyrophosphate buffer, pH 7.0, and six cell pellets representing approximately 6 L each of water were stored at −80°C.

**Table 1:** **Percentages of land cover of hydrological units sampled**.

Site^*^	**Distance (km)^†^**	**Developed, open**	**Developed, low**	**Developed, med**	**Developed, high**	**Sum developed^‡^**	**Dedciduous forest**	**Evergreen forest**	**Mixed forest**	**Sum forest^§^**	**Pasture**	**Cultivated**	**Sum agriculture^¶^**
Itasca^a^	0	2.51	0.65	0.17	0.08	3.42	34.50	9.99	4.23	48.72	5.65	1.01	6.66
St. Cloud^b^	263	5.95	1.95	1.26	0.40	9.56	16.45	1.11	0.05	17.61	16.96	38.93	55.89
Clearwater^b^	271	5.95	1.95	1.26	0.40	9.56	16.45	1.11	0.05	17.61	16.96	38.93	55.89
Twin Cities^c^	311	8.57	14.22	6.28	2.16	31.22	14.13	1.14	0.05	15.32	16.58	11.40	27.99
MN River^d^	NA	4.97	4.95	2.70	1.23	13.86	7.50	0.20	0.02	7.73	10.45	58.49	68.94
Confluence^c^	313	8.57	14.22	6.28	2.16	31.22	14.13	1.14	0.05	15.32	16.58	11.40	27.99
Hastings^c^	330	8.57	14.22	6.28	2.16	31.22	14.13	1.14	0.05	15.32	16.58	11.40	27.99
St. Croix River^e^	NA	4.52	1.62	0.61	0.20	6.95	27.01	2.07	0.54	29.62	23.73	22.05	45.78
Red Wing^f^	362	5.32	3.47	1.64	0.44	10.87	19.06	0.21	0.05	19.32	12.21	43.94	56.14
LaCrescent^g^	401	3.76	4.24	1.73	0.42	10.14	40.46	1.92	0.15	42.53	10.95	22.05	33.00
Zumbro River^h^	NA	5.72	2.37	0.70	0.25	9.04	9.59	0.12	0.01	9.71	11.42	55.66	67.07

* *Samples with the same superscript (a–h) are within the same HUC boundaries*.

† *Distance from the headwaters. Samples marked NA are major confluent rivers*.

‡ *Sum of all developed (open—high) area*.

§ *Sum of deciduous, evergreen, and mixed forest area*.

¶ *Sum of pasture and cultivated land*.

### DNA extraction and sequencing

DNA was extracted from two replicate cell pellets per site using the DNA Isolation Kit for Water (Epicentre, Madison, WI). The V6 hypervariable region of the 16S rDNA was amplified using the 967F/1046R barcoded primer set (Sogin et al., 2006), and amplicons were gel-purified using the QiaQuick® Gel Extraction Kit (Qiagen, Valencia, CA). Replicate sequence data was generated by paired-end sequencing of purified amplicon pools on the Illumina MiSeq (DNA from one cell pellet, 2 × 150 bp read length) and HiSeq2000 (DNA from the second cell pellet, amplicons sequenced in duplicate at 2 × 100 bp). Duplicate sequencing was performed because a third cell pellet was not available for additional DNA extraction resulting from use of pellets in other experiments not described here. Interpretations of sequence data have been previously shown to be reproducible across platforms (Caporaso et al., 2012), and use of the HiSeq2000 enabled more efficient multiplexing of samples. Sequencing was performed by the University of Minnesota Genomics Center. Sequences were deposited to the GenBank Sequence Read Archive under accession number SRP018728.

### Metadata collection

Physicochemical parameters, including temperature, pH, and rainfall up to 72 h prior to sampling was collected at the time of sampling. In addition, two additional 1 L water samples were collected in sterile amber bottles and stored at 4°C for further chemical analyses. *Escherichia coli* concentration was determined by membrane filtration and plating on mTEC agar using standard methods (United States Environmental Protection Agency, 2002) and data are expressed as colony-forming units (CFU) per 100 ml. Concentrations of ammonium (NH_4_), nitrite/nitrate (NO_2_/NO_3_), total phosphorus (total-P), orthophosphate, total organic carbon (carbon), and total dissolved solids (TDS) were measured by the Research Analytical Laboratories (RAL; University of Minnesota, St. Paul, MN). In addition, inductively coupled plasma mass spectrometry (ICP) analysis was used to quantify ion concentrations, and quantification of concentrations of various xenobiotic compounds, classified as antibiotics, endocrine disruptors, pharmaceuticals, personal care products, and pesticides was performed by the RAL using liquid chromatography-mass spectrometry. Analytes were exhaustively selected based on the capability of the RAL, and xenobiotic compounds measured were determined by RAL capabilities. All physical parameters and analytes measured are shown in Table 2.

**Table 2:** **Summary of analytes measured among all sampling sites during both years of study**.

**Class**	**Analyte**	**2011**				**2012**			
		**Mean**	***SD***	**Min**	**Max**	**Mean**	***SD***	**Min**	**Max**
Bacteria (CFU 100 ml^−1^)	*E. coli*	76.82	105.28	5.00	300.00	15.05	27.16	ND^*^	93.50
Physical parameters	Temperate (°C)	18.18	2.32	12.00	21.00	21.52	1.76	18.20	23.80
	pH	7.72	0.28	7.20	8.10	7.59	0.30	6.89	7.89
	Cumulative Rainfall (mm)	7.39	7.54	0.00	21.85	13.44	14.84	0.00	43.69
	72 h Rainfall (mm)	1.78	2.73	0.00	7.37	4.66	8.29	0.00	21.84
	48 h Rainfall (mm)	3.05	4.85	0.00	11.18	3.53	11.26	0.00	38.61
	24 h Rainfall (mm)	2.56	5.72	0.00	17.53	5.24	6.67	0.00	17.78
Nutrients (mg L^−1^)	Organic carbon	6.20	1.92	1.59	8.99	8.72	3.58	2.06	14.47
	Ammonium	0.07	0.02	0.05	0.11	0.05	0.02	0.04	0.09
	Nitrate/nitrite	2.50	2.36	0.05	7.57	1.97	1.97	0.16	6.19
	Total phosphorus	0.19	0.06	0.08	0.28	0.11	0.01	0.09	0.13
	Orthophosphate	0.05	0.02	0.02	0.09	0.06	0.03	0.02	0.13
	TDS^†^	79.88	32.39	21.87	139.42	44.19	18.51	13.35	72.13
Ions or Metals (mg L^−1^)	Al	0.13	0.11	ND	0.41	0.42	0.30	0.08	1.18
	B	0.05	0.03	0.02	0.10	ND	ND	ND	ND
	Ca	65.43	25.93	17.79	112.79	36.72	14.17	11.50	57.75
	Cd	ND	ND	ND	ND	0.02	ND	0.02	0.02
	Cr	ND	0.01	ND	0.02	ND	ND	ND	ND
	Cu	0.06	0.16	ND	0.57	ND	ND	ND	0.01
	Fe	0.30	0.15	0.09	0.49	0.86	0.44	0.04	1.59
	K	1.97	0.89	0.90	3.48	2.17	0.83	0.79	3.52
	Mg	26.19	14.10	6.39	55.54	13.54	6.37	3.63	25.00
	Mn	0.05	0.02	0.02	0.08	0.06	0.02	0.02	0.09
	Na	11.75	5.48	3.62	21.39	5.44	2.72	1.63	9.91
	Ni	ND	ND	ND	ND	ND	ND	ND	ND
	P	ND	ND	ND	ND	0.97	2.20	0.08	7.79
	Pb	ND	ND	ND	ND	ND	ND	ND	ND
	Zn	0.02	0.03	ND	0.10	0.10	0.06	0.03	0.21
Antibiotics (ng ml^−1^)	Erythromycin	ND	ND	ND	ND	ND	ND	ND	ND
	Monensin	ND	ND	ND	ND	ND	ND	ND	ND
	Sulfumathoxazole	ND	ND	ND	ND	ND	ND	ND	ND
Pesticides (ng ml^−1^)	Acetochlor	9.59	15.10	ND	43.80	67.01	72.50	ND	231.00
	Atrazine	2.71	4.38	ND	14.30	4.51	1.42	3.07	7.24
	Carbaryl	ND	ND	ND	ND	0.61	1.05	0.26	3.89
	D-atrazine	NA^‡^	NA	NA	NA	ND	ND	ND	ND
	Iprodione	ND	ND	ND	ND	ND	ND	ND	ND
	Metolachlor	ND	ND	ND	ND	141.64	132.06	ND	374.00
Pharmaceuticals (ng ml^−1^)	Acetaminophen	ND	ND	ND	ND	ND	ND	ND	ND
	Caffeine	4.49	3.65	ND	11.60	10.28	7.93	3.50	32.70
	Ibuprofen	ND	ND	ND	ND	ND	ND	ND	ND
Endocrine disrupters (ng ml^−1^)	4-Nonylphenol	ND	ND	ND	ND	ND	ND	ND	ND
	Daidzein	ND	ND	ND	ND	ND	ND	ND	ND
	Carbamazepine	ND	ND	ND	ND	0.56	1.81	ND	6.20
	Fomonentin	ND	ND	ND	ND	ND	ND	ND	ND
	Genistein	ND	ND	ND	ND	ND	ND	ND	ND
	meta-Chlorophenylpiperazine	28.77	52.97	ND	162.00	ND	ND	ND	ND
	Zeranol	ND	ND	ND	ND	ND	ND	ND	ND
Personal care products (ng ml^−1^)	Cotinine	1.35	0.71	ND	2.56	ND	ND	ND	ND
	DEET	ND	ND	ND	ND	5.77	7.74	ND	21.40

* *ND: Analyte was not detected*.

† *Total dissolved solids*.

‡ *NA: Analyte was not measured*.

Land cover data was extrapolated from the National Land Cover Database (NLCD 2006) (Fry et al., 2011) by overlaying a map of hydrologic unit code (HUC) boundaries (1:250,000 scale) using ArcGIS (Esri, Redlands, CA) and expressed as a percentage of the total area of the HUC boundary (Table 1). Maps were obtained from the United States Geological Survey [http://water.usgs.gov/maps.html]. NLCD codes for similar land cover types (e.g., “developed, low” and “developed, med”) were summed in order to evaluate the influence of major land cover types. Major land cover categories investigated were “developed” (urban anthropogenic impacts), “forested” (unimpacted by anthropogenic activity), and “agricultural” (agricultural anthropogenic impacts). Agricultural land throughout this manuscript refers to the sum of pastureland and cultivated (crop) land, while “pasture” specifically references pastureland alone.

### Sequence processing

All sequence processing was performed using mothur software ver. 1.29.2 and 1.32.0 (Schloss et al., 2009). Sequences were trimmed to 100 nt, paired-end aligned using fastq-join (Aronesty, 2013), and screened for quality. Sequences that had a quality score <35 over a window of 50 nt, had a mismatch to a primer or barcode sequence, had homopolymers >8 nt, or had an ambiguous base (N) were excluded from analysis. Singleton sequences were removed in mothur and chimeras were removed using UCHIME (Edgar et al., 2011). The number of sequence reads in each sample was normalized by random subsampling to 25,703 sequence reads per sample. Sequences were aligned against the SILVA database ver. 102 (Pruesse et al., 2007), OTUs were clustered using the furthest-neighbor algorithm at 97% similarity, and OTUs were classified against the Ribosomal Database Project ver. 9 database (Cole et al., 2009).

### Statistical analyses

All diversity calculations, ordination plots, and community comparisons were performed using mothur (Schloss et al., 2009) and Bray-Curtis dissimilarity matrices (Bray and Curtis, 1957). For all analyses, unless otherwise stated, replicates were maintained as separate samples and grouped by using various design files. Three analyses were used to evaluate differences in community structure among sampling sites: (i) Beta-diversity differences between sites were determined using UniFrac metrics (Lozupone and Knight, 2005), which take into account raw phylogenetic differences between sets of taxa (unweighted) or abundance-weighted phylogenetic differences (weighted); (ii) analysis of similarity (ANOSIM) (Clarke, 1993), in which rank order differences in community structure are evaluated from a dissimilarity matrix was performed, and (iii) analysis of molecular variance (AMOVA) (Excoffier et al., 1992), which is similar to a non-parametric analysis of variance (ANOVA) utilizing dissimilarity matrices was also performed. All community-level analyses were also done using mothur and statistics were calculated using 1000 iterations. Spearman correlations, ANOVA, generalized linear modeling, and discriminant function analysis (DFA) were performed using SPSS Statistics software ver. 19.0 (IBM, Armonk, NY). All statistics were evaluated at α = 0.05.

### Network modeling

For network modeling, bacteria were classified to orders, as this level of taxonomic resolution has been used previously for interrogating associations among bacterial communities (Gilbert et al., 2012), and the accuracy of more specific classification (e.g., to family or genus) may be unreliable with short sequence reads used here (Mizrahi-Man et al., 2013). Local similarity analyses were performed using the eLSA software package (Xia et al., 2011) with no time-delay [-d 0], normalization to both percentile and Z-score [-n percentileZ] and replicates were averaged (default setting). A total of 191 parameters were included in the LSA model; parameters that were not detected during both years of study were excluded. LSA results were visualized using Cytoscape ver 3.0.2 (Shannon et al., 2003).

For Bayesian network inference, environmental parameters were normalized as described previously (Larsen et al., 2012) using the equation $Env\_norm_{i}^{\;j}=\frac{\left(MAX\left(Env^{\;j}\right)-\;Env_{i}^{\;j}\right)}{\left(MAX\left(Env^{\;j}\right)-\;MIN\left(Env^{\;j}\right)\right)}\times 99+\;1$. Environmental parameters were incorporated into a single input matrix with the 15 most abundant bacterial orders, and for simplicity, reported as the mean percentages of relative abundance among triplicates (a number between 0 and 100). Relationships were inferred using the Bayesian Network Inference with Java Objects (BANJO) ver. 2.2.0 (Smith et al., 2006), and settings used were similar to those previously described (Larsen et al., 2012). Networks were considered using simulated annealing and the All Local Moves proposer, with a maximum of five parents. Only environmental parameters were considered as parents. Only significant associations (at α = 0.05) were incorporated into the final consensus network of highest-scoring networks inferred and were visualized using Cytoscape software.

## Results

### Bacterial community composition

**Figure 1:**
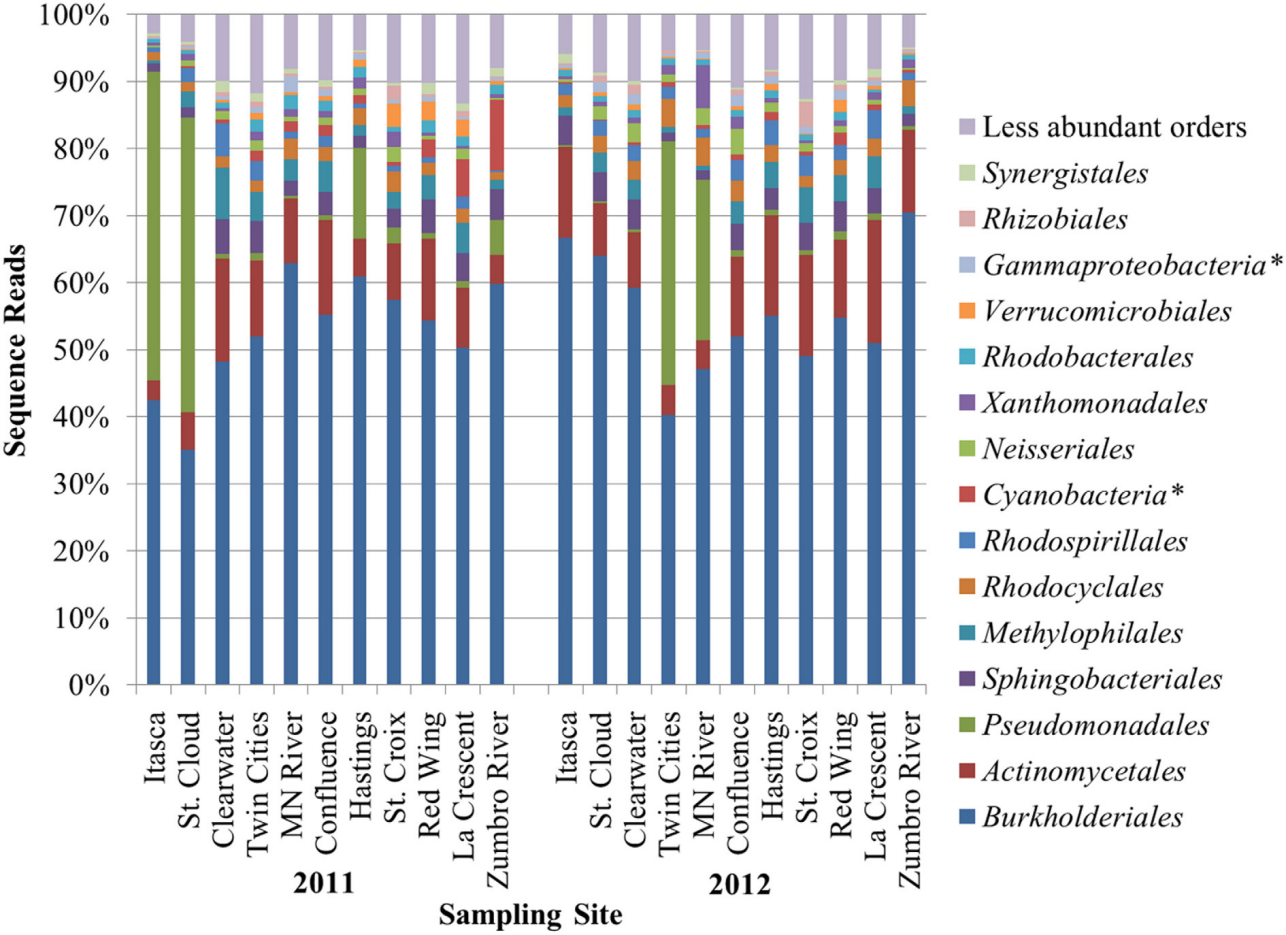
**Distribution of the most abundant orders identified at sampling sites among triplicate samples at each site**. Asterisks (^*^) denote orders designated *incertae sedis*.

**Figure 2:**
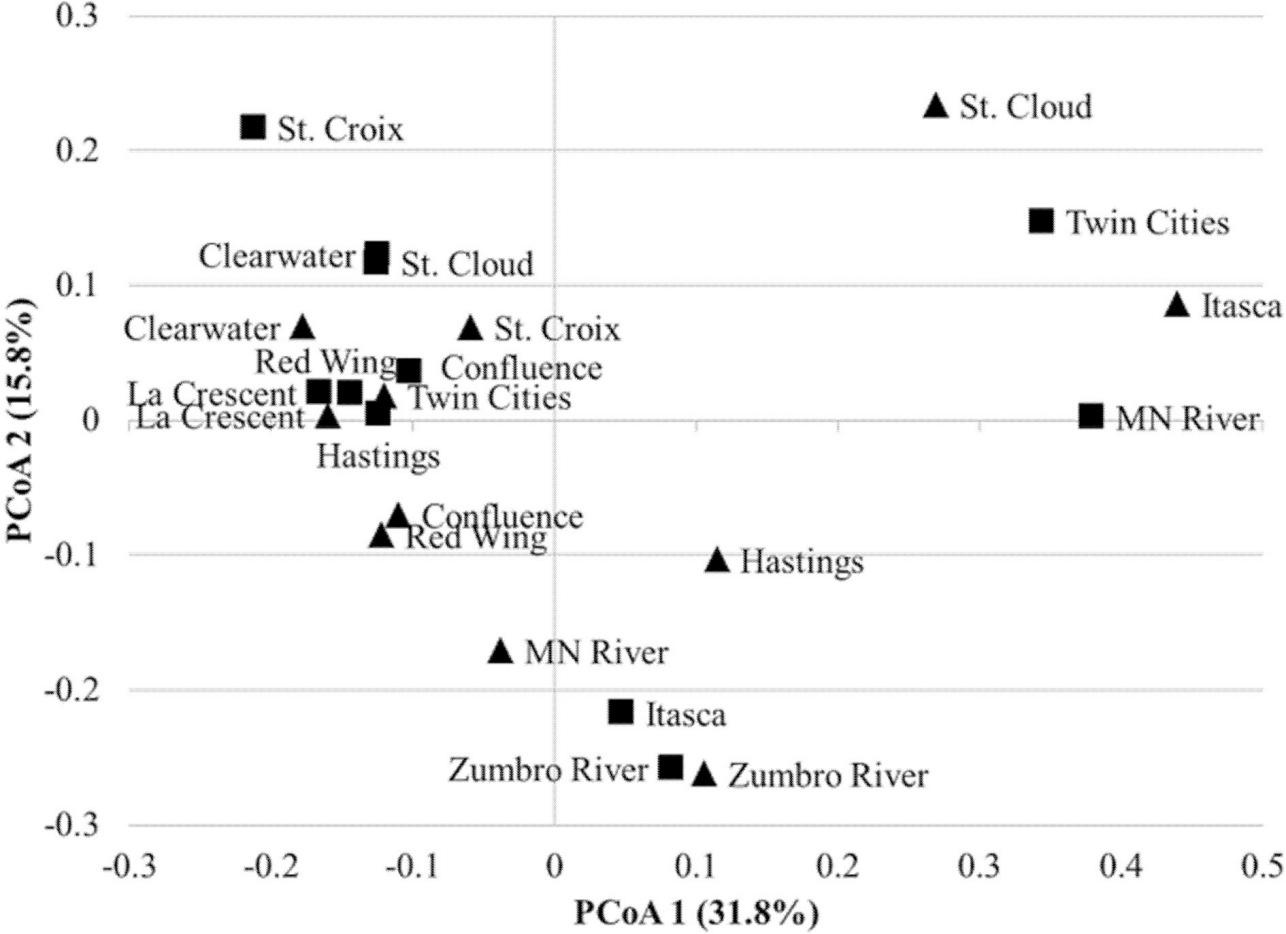
**Principal coordinate analysis of bacterial communities from 2011 (▴) to 2012 (∎) samples (*r*^2^ = 0.848)**. All replicates were merged for ordination only; statistics were calculated with replicates separated. A total of 20 axes were necessary to explain all variation.

A mean of 1450 ± 266 OTUs were identified among all samples and could be classified to 153 orders. An average of 0.02 ± 0.02% of sequence reads could not be classified to an order among all replicate samples. Differences in alpha diversity were not significantly different between years (mean Shannon index 4.02 ± 0.50, *P* = 0.128). The bacterial community composition of all samples was predominantly comprised of the orders *Burkholderiales* (54.0% mean abundance), *Actinomycetales* (10.1%), *Pseudomonadales* (8.3%), *Sphingobacteriales* (3.4%), *Methylophilales* (3.1%), *Rhodocyclales* (2.4%), and *Rhodospirillales* (2.0%; Figure 1). All other orders accounted for a mean of <2.0% of sequence reads. At two sites sampled in either year (Itasca and St. Cloud in 2011 and Twin Cities and Minnesota River in 2012), a majority of sequence reads classified to *Pseudomonadales*, and this result was consistent among replicate sequence data at these sites. While the dominant orders were generally consistent among samples, bacterial communities differed significantly between samples collected in 2011 and 2012 (unweighted and weighted UniFrac *P* < 0.001, ANOSIM *P* = 0.007, AMOVA *P* = 0.007; Figure 2).

### Local similarity analysis

**Figure 3:**
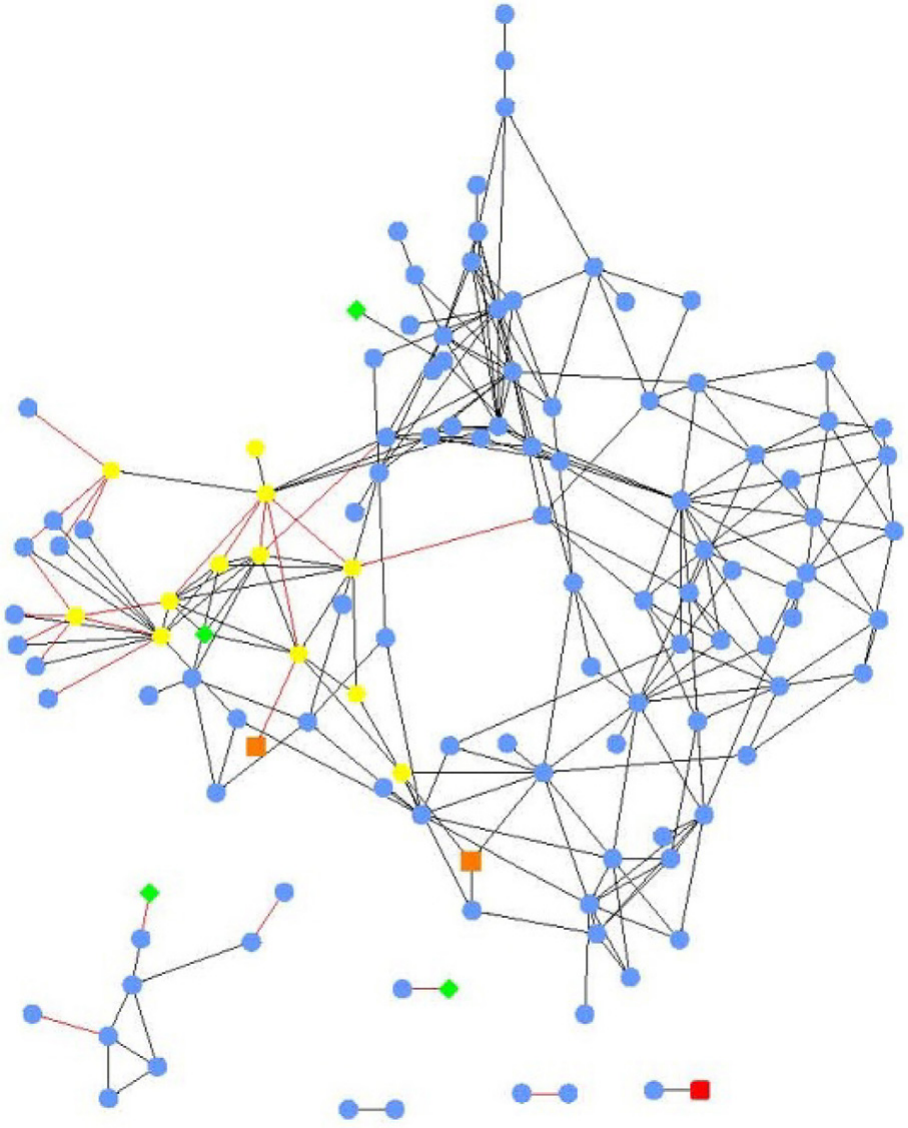
**Local similarity analysis network of bacterial orders (blue circles), physicochemical parameters (green diamonds), chemicals (nutrients and ions; yellow hexagons), xenobiotic compounds (red squares), and land use (orange squares)**. All relationships were significant (*P* < 0.05, *q* < 0.003). Black edges indicate positive local similarity scores and red edges are negative, length is arbitrary.

Local similarity analysis revealed that the relative abundances of bacterial orders were generally more significantly intercorrelated amongst themselves, than with nutrient or chemical concentrations (Figure 3). Importantly, *E. coli* concentration was not significantly associated with other analyte concentrations or abundances of specific orders. Specific and stronger associations (−0.7 > Spearman's *r* > 0.7) among all parameters and bacterial orders included in LSA are shown in Supplementary Figures S1, S2.

Among the nutrient concentrations measured, only the amounts of organic carbon and TDS were significantly associated with order abundances. Organic carbon concentration was associated with the abundances of *Acidobacteria* Group 2, *Gemmatimonadales*, and *Sneathiellales* (Spearman's *r* = 0.640, 0.631, and 0.792), while TDS concentration was inversely correlated with abundance of *Acidobacteria* Group 2 (*r* = −0.709). Among ions measured, the concentration of potassium was significantly associated with abundances of *Puniceicoccales, Thiotrichales*, and *Verrucomicrobia* Subdivision 5 (*r* = 0.624, 0.584, and 0.761).

Developed land use was also associated with potassium concentration (*r* = 0.730) as well as *Thiotrichales* and *Verrucomicrobia* Subdivision 5 abundances (*r* = 0.734 and 0.715). Magnesium concentration was negatively correlated with abundance of *Anaerolineales* (*r* = −0.556), and boron concentration was positively correlated with abundances of *Rhodobacterales* (*r* = 0.534) and several rare orders for which correlation coefficients could not be obtained. Several of these rare orders were also negatively associated with iron and zinc concentrations. Among the xenobiotic chemicals tested, only the concentration of acetochlor was significantly associated with *Acidobacteria* Group 1 (*r* = 0.766).

### Association of physical and chemical parameters with community composition

Correlations among nutrient and chemical concentrations with community diversity and order abundances were also observed regionally via traditional correlation analyses. Similar to LSA, however, significant intercorrelations were also observed between analyte concentrations, making the biological importance of associations with community composition difficult to interpret.

Temperature and cumulative, 3-day antecedent rainfall were significantly higher in 2011 than 2012 (*P* = 0.041 and < 0.001, respectively; Table 2). Among all the nutrients measured, organic carbon, ammonium, total phosphorous, and TDS were significantly greater in 2011 vs. 2012 (*P* ≤ 0.001). Over both years of the study, temperature and organic carbon concentrations were positively correlated with diversity, as measured by the Shannon index (*r* = 0.292 and 0.369, *P* = 0.018, and 0.002, respectively) while pH, 48 h antecedent rainfall, and TDS concentration were inversely correlated (*r* = −0.325, −0.462, and −0.246, *P* = 0.008, <0.001, and 0.046, respectively). A generalized linear model was constructed to relate pH and nutrient concentrations to Shannon diversity. Temperature and rainfall were excluded as these were not associated with land cover. Concentrations of organic carbon and nitrite/nitrate were found to have significant main effects on Shannon diversity (*P* = 0.009 and 0.045, respectively), although the abundances of most of the orders were significantly correlated with the concentration of at least one of the nutrients measured (*P* < 0.05). Supplementary Figures S3, S4 show a subset of these orders that also had relative abundances related to land coverage.

The concentrations of the majority of ions also differed significantly (*P* < 0.05) between years, except for Cr, Cu, K, and Mn. The concentrations of Al, Mn, and K were significantly correlated with richness measured as the number of OTUs identified (*r* = 0.294–0.310, *P* ≤ 0.016), while the concentrations of Ca were negatively correlated to the Shannon diversity index (*r* = −0.246, *P* = 0.047). However, the concentration of N,N-Diethyl-meta-toluamide (the insecticide DEET) significantly increased richness (*r* = 0.283, *P* = 0.021), while the concentration of carbaryl was inversely correlated (*r* = −0.288, *P* = 0.019) to richness by traditional analysis.

### Intercorrelation of water quality parameters

Among parameters traditionally used to evaluate water quality, traditional bivariate correlation analysis indicated that the concentration of *E. coli* was only significantly correlated with total phosphorus and TDS concentrations (*r* = 0.527 and 0.328, *P* < 0.001 and 0.007, respectively) and inversely correlated with organic carbon concentrations (*r* = −0.363, *P* = 0.003). Nitrite/nitrate, orthophosphate, and TDS concentrations were all positively correlated with each other (*r* = 0.665–0.850, *P* < 0.001), and negatively correlated with organic carbon concentration (*r* = −0.546 to −0.804, *P* < 0.001). Ammonium, total phosphorus, and TDS concentrations were also significantly positively correlated with each other (*r* = 0.261–0.353, *P* ≤ 0.035). Negative correlations between ammonium or phosphorus concentrations and organic carbon concentrations were not significant at α = 0.05.

### Association of nutrient and chemical concentrations with land cover

To simplify the interpretation of intercorrelations among analytes and land cover, analyte concentrations were related to major land coverage categories observed (developed, forested, or pasture) by traditional bivariate correlation analysis (Table 3). Pastureland alone was evaluated because cultivated land was poorly correlated with the relative abundances of nearly every order, and a negative correlation between pastureland and *E. coli* concentration was significant (*r* = −0.303, *P* = 0.013). Generally, a greater percentage of developed land area was associated with higher pH and increased nitrite/nitrate, orthophosphate, and TDS concentrations, as well as concentrations of several ions. In contrast, the concentrations of these parameters tended to decrease with greater percentages of forested or pasture land within the HUC boundary (Table 3). A greater percentage of pastureland within a hydrologic unit was also well correlated with increased organic carbon concentration (*r* = 0.695, *P* < 0.001). Concentrations of some pesticides, endocrine disrupters, and personal care products were also negatively correlated with the percentage of forested area within a hydrologic unit (*r* = −0.279 to −0.375, *P* ≤ 0.033), while acetochlor concentration was positively correlated with developed area (*r* = 0.421, *P* < 0.001).

**Table 3:** **Correlation coefficients relating analyte concentrations with major land coverage patterns observed**.

**Class**	**Analyte**	**Developed**	**Forested**	**Pasture**
Bacterium	*E. coli*	−0.090 (0.472)	0.108 (0.389)	−0.303 (0.013)^*^
Physical Parameters	pH	**0.379 (0.002)**	**−0.577 (<0.001)**	**−0.443 (<0.001)**
Nutrients	Carbon	0.104 (0.406)	**0.246 (0.046)**	**0.695 (<0.001)**
	Ammonium	0.154 (0.218)	0.030 (0.812)	0.161 (0.197)
	Nitrate/nitrite	**0.412 (0.001)**	**−0.657 (<0.001)**	**−0.384 (0.001)**
	Total phosphorus	0.023 (0.853)	−0.089 (0.479)	0.071 (0.570)
	Orthophosphate	**0.497 (<0.001)**	**−0.532 (<0.001)**	**−0.412 (0.001)**
	TDS	**0.312 (0.011)**	**−0.518 (<0.001)**	**−0.465 (<0.001)**
Ions	Al	**0.297 (0.015)**	**−0.397 (0.001)**	−0.137 (0.274)
	B	0.214 (0.085)	−0.099 (0.427)	−0.047 (0.711)
	Ca	**0.282 (0.022)**	**−0.457 (<0.001)**	**−0.482 (<0.001)**
	Cd	0.000 (1.000)	0.000 (1.000)	0.000 (1.000)
	Cr	0.070 (0.575)	0.140 (0.264)	−0.073 (0.563)
	Cu	**−0.399 (0.001)**	**0.421 (<0.001)**	0.116 (0.354)
	Fe	0.118 (0.346)	−0.152 (0.222)	0.225 (0.070)
	K	**0.721 (< 0.001)**	**−0.637 (<0.001)**	**−0.253 (0.041)**
	Mg	**0.381 (0.002)**	**−0.486 (<0.001)**	**−0.476 (<0.001)**
	Mn	**0.292 (0.017)**	−0.71 (0.569)	**0.261 (0.035)**
	Na	**0.469 (<0.001)**	**−0.497 (<0.001)**	−0.232 (0.060)
	P	0.001 (0.992)	0.023 (0.853)	−0.090 (0.470)
	Zn	−0.173 (0.165)	**0.391 (0.001)**	0.046 (0.715)
Pesticide	Acetochlor	**0.421 (<0.001)**	−0.146 (0.243)	−0.220 (0.076)
	Atrazine	0.234 (0.058)	**−0.375 (0.002)**	−0.093 (0.456)
	Carbaryl	−0.041 (0.745)	−0.132 (0.290)	0.001 (0.992)
	Metolachlor	0.238 (0.054)	**−0.283 (0.021)**	0.038 (0.764)
Pharmaceutical	Caffeine	0.075 (0.549)	0.017 (0.895)	0.008 (0.947)
Endocrine disrupter	Carbamazepine	−0.211 (0.089)	**−0.279 (0.023)**	−0.181 (0.145)
Personal care products	Cotinine	0.190 (0.126)	0.028 (0.826)	0.137 (0.272)
	DEET	0.157 (0.207)	**−0.263 (0.033)**	**0.244 (0.049)**

* *P-values are shown in parentheses. Bold values indicate statistically significant correlations*.

### Association of land coverage with community structure

Sites were grouped by land coverage category (developed, forested, or combined agricultural) based on the greatest percentage of land coverage within the HUC boundary in which the site was located (Table 1). Changes in community membership (differences in phylogenetic branching) and relative abundance of taxa were significantly different (*P* ≤ 0.008) among land coverage categories (both years combined), as evaluated by comparing unweighted and weighted UniFrac metrics, respectively. Similarly, these differences were found to be significant (*P* = 0.037) in individual years, except when using unweighted UniFrac metrics of differences between the developed and forested categories in 2012 (*P* = 0.066). Within a land coverage category, however, differences in phylogenetic structure did not differ significantly among sampling sites from either year (*P* ≥ 0.200), but the majority of pairwise comparisons showed significant differences when relative abundance of taxa was considered at α = 0.05.

However, despite phylogenetic differences found by evaluating UniFrac values, β-diversity was not significantly different (*P* = 0.345) among land coverage categories when evaluated using ANOSIM over both years or during a single year (*P* = 0.359 and 0.237 for 2011 and 2012, respectively). Furthermore, clustering of sites by primary surrounding land-coverage category was not significant in 2011 or 2012 (AMOVA *P* = 0.205 and 0.101, respectively).

Richness, determined as the number of OTUs, was significantly higher at sites surrounded by developed land than those surrounded by forest or agriculture (*P* = 0.001). Shannon diversity was significantly higher at developed sites compared to forested sites (*P* = 0.017), but the difference in diversity between developed and agricultural sites was not significant by *post-hoc* test (*P* = 0.371). The percentages of total developed and pasture land coverage, but not total agricultural land coverage, within an HUC boundary were significantly correlated with richness (number of OTUs observed, *r* = 0.583, *P* < 0.001; *r* = 0.261, *P* = 0.034; respective to coverage type) and Shannon indices (*r* = 0.334, *P* = 0.006; *r* = 0.463, *P* < 0.001) by traditional bivariate correlation analysis. Taxonomic orders that were ≥0.05% of sequence reads (on average) among all samples (*n* = 51) were evaluated by traditional analysis to determine associations with the predominant surrounding land coverage. Thirty-seven orders were correlated with the percentage of at least one of these land use types (Supplementary Figures S3, S4).

### Bayesian modeling of community variation

**Figure 4:**
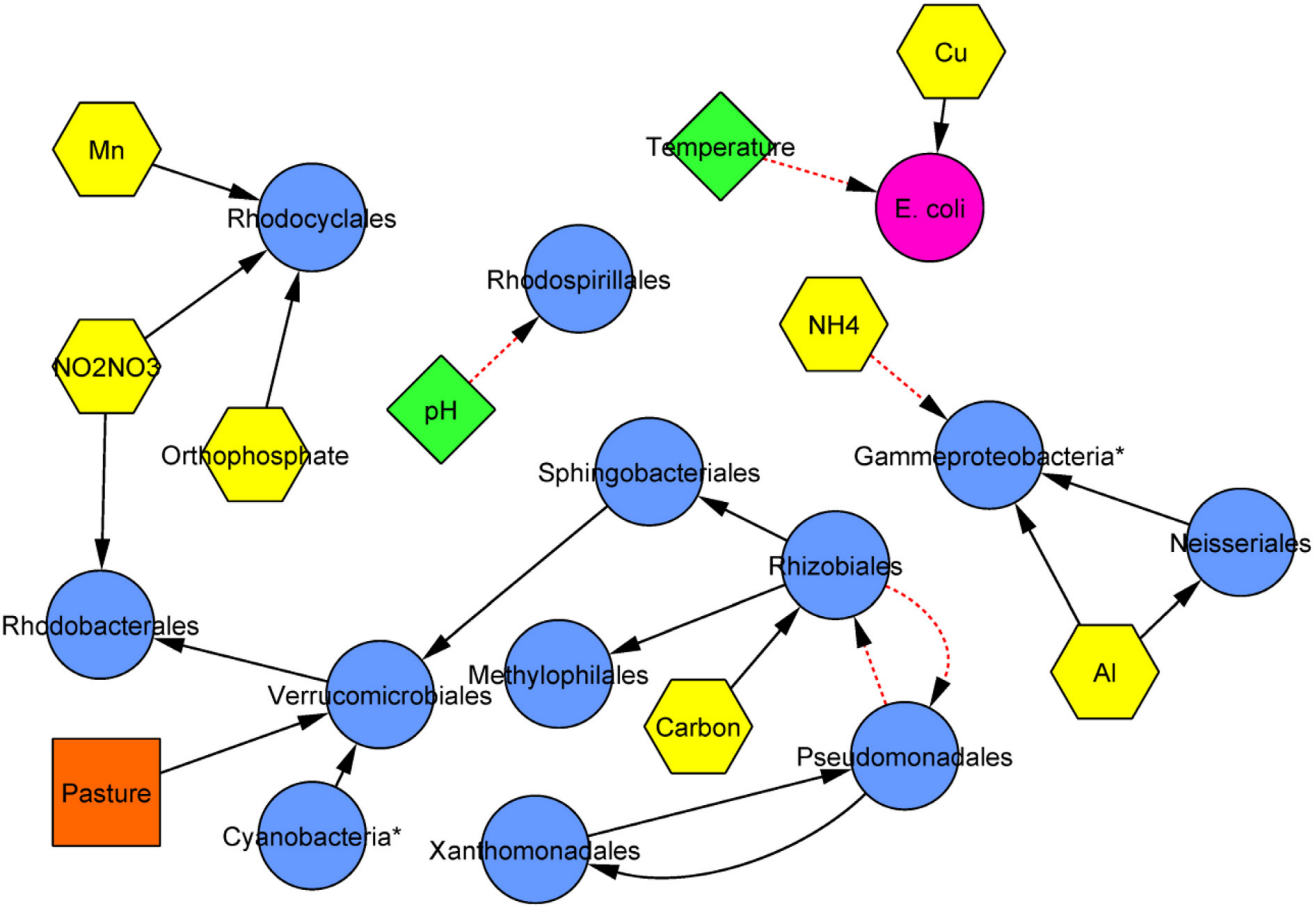
**Consensus inferred Bayesian network relating bacterial orders (blue circles), physicochemical parameters (green diamonds), chemicals (nutrients and ions; yellow hexagons), *E. coli* (pink circle), and land use (orange squares)**. Solid black lines indicate positive associations while dashed red lines indicate negative associations and arrows are directed from parent to child. Edge length is arbitrary. Asterisks (^*^) designate orders that are unclassified or designated *incertae sedis*.

Modeling by Bayesian inference was performed to elucidate potentially biologically important parameters influencing community composition among analytes and land cover measured. Bayesian inference revealed that nine analytes and the percentage of pasture coverage were significantly and directionally associated with the relative abundance of 12 of 15 (80%) of the most abundant taxonomic orders (Figure 4). Interestingly, the concentration of *E. coli* at sites was not related to the relative abundance of any of these orders. However, among the nutrients examined, the concentrations of ammonium, organic carbon, nitrite/nitrate, and orthophosphate were all associated with the relative abundance of at least one order. Furthermore, while directional relationships among specific orders could be inferred, they did not include the two most abundant orders identified—*Burkholderiales* and *Actinomycetales*.

### Discriminant function analysis (DFA) of land cover

The DFA revealed two discriminant functions (i.e., linear functions in which order abundances were weighted such that land coverage types are maximally dispersed) to explain variance in major land coverage types (*P* < 0.001 for both functions; DFA1 canonical coefficient = 0.994, Wilks' λ = 0.027; DFA 2 canonical coefficient = 0.871, Wilks' λ = 0.242). Fourteen orders were identified that were best related to the primary surrounding land coverage of samples collected during both years (orders and coefficients, the absolute values of which indicate the effect size, are shown in Table 4). Of these orders, 7 of 14 (50%) showed differences in relative abundance over both years of study between the three major land-coverage types, via Tukey's *post-hoc* test (Table 4). Among the orders that were significantly more abundant at sites with primarily developed land coverage, only *Aeromonadales* and *Nitrosomonadales* were significantly more abundant compared to primarily agricultural sites (*P* ≤ 0.005). Among those more abundant at forested sites, only the *Gammaproteobacteria* order was also greater when compared against agricultural sites (*P* = 0.019).

**Table 4:** **Standardized canonical discriminant function coefficients for taxonomic orders best associated with land coverage type by DFA**.

**Taxonomic order**	**DFA1 (72.2%)**	**DFA2 (27.8%)**
*Methylophilales*	1.797	1.242
*Cyanobacteria* (unclassified)	0.652	0.705
Rhodobacterales^*^	2.314	0.097
*Gammaproteobacteria incertae sedis*^†^	−1.357	0.603
*Rhizobiales*	0.058	0.688
*Synergistales*^†^	−3.667	−1.091
*Chromatiales*	0.976	0.924
*Bacillales*	1.266	1.218
*Verrucomicrobia* Subdivision3 genera incertae sedis^*^	0.502	−1.461
*Prolixibacter*^†^	0.877	−0.137
Aeromonadales^*^	0.697	−0.815
*Legionellales*	−2.333	−1.290
*Bacteroidales*	−0.005	1.680
Nitrosomonadales^*^	0.308	−1.486

* *Order was significantly more abundant at sites with primarily developed land coverage (P ≤ 0.036)*.

† *Order was significantly more abundant at sites with primarily forested land coverage*.

To better resolve members of the community that might indicate inputs from specific land cover types, the sequences of representative OTUs from each order that showed a significant association with land cover were compared manually against the GenBank non-redundant (nr/nt) database via blastn search (BLAST). Representative OTUs were the most abundant OTUs in each order and generally had 10- to 100-fold more sequence reads than all other OTUs classified to that order over the entire dataset. The isolation sources of top-matches were taken from the submitters' annotations within GenBank metadata. In general, top BLAST matches for orders that were more abundant at forested sites tended to be strictly from freshwater habitats while those more abundant at developed sites were associated with wastewater treatment or oil contamination (Table 5).

**Table 5:** **Identification of representative OTUs via BLAST search**.

**Order**	**Species ID**	**Isolation sources**
*Rhodobacterales*	Uncultured *Rhodobacter* sp.	Oilfield-produced water
	Uncultured bacterium	Wastewater treatment plant biofilter
	Uncultured bacterium	Oil-contaminated groundwater
	*Roseinatronobacter* sp.	Alkaline hypersaline lake
	Uncultured bacterium	Water; Hungary
*Gammaproteobacteria incertae sedis*	Uncultured bacterium (2)	Big Lake; Mljet, Dubrovnik, Croatia
	Uncultured Methylotenera sp.	Holocene marine sediment
	Uncultured betaproteobacterium	Freshwater biofilm
	Uncultured bacterium	Municipal drinking water system, raw water influent
*Synergistales*	Uncultured bacterium (2)	Arctic thaw pond water
	Uncultured bacterium (2)	Lake Mizugaki; Yamanashi, Japan
	Uncultured bacterium	Deep-water sponge (*Baikalospongia intermedia*)
*Verrucomicrobia* Subdivision 3 *incertae sedis*	Uncultured bacterium	Municipal drinking water system, tap water
	Uncultured bacterium	Water, 17th Street Canal, New Orleans, LA, USA
	Uncultured bacterium	Lake Poyang; China
	Uncultured bacterium	Soil; Harvard Forest, MA, USA
	Uncultured bacterium	Soil; Adulam, Israel
*Prolixibacter*	Uncultured bacterium	Lake Mizugaki; Yamanashi, Japan
	Uncultured bacterium	Municipal drinking water system, raw water influent
	Uncultured bacterium	Amazon River, Brazil
	Uncultured bacterium	Las Cumbres Lake; Panama
	Uncultured bacterium	Diseased leaf; Lake Taihu; China
*Aeromonadales*	*Aeromonas veronii*	Pond loach (*Misgurnus anguillicaudatus*)
	*Aeromonas salmonicida* (2)	Channel catfish (*Ictalurus punctatus*)
	*Aeromonas dhakensis*	Membrane bioreactor activated sludge
	*Aeromonas hydrophila*	Membrane bioreactor activated sludge
*Nitrosomonadales*	Uncultured bacterium	Yong Ding River; Beijing China
	Uncultured bacterium	Typha rhizosphere; Bai River; Beijing China
	Uncultured bacterium	Municipal drinking water distribution system
	Uncultured bacterium	Activated sludge
	Uncultured betaproteobacterium	Activated sludge

*Results of the top 5 BLAST matches are shown and numbers in parenthesis indicate the number of hits to multiple isolates from the same source*.

## Discussion

In this study, we evaluated the relationships between environmental and chemical parameters and the abundances of bacterial orders and observed that throughout the river at large, the abundances of bacterial orders were associated with regional variation in nutrient and chemical concentrations that are intercorrelated with land coverage patterns. The relationship between land cover and nutrient concentrations has been frequently reported (Gilbert et al., 2009; Fortunato and Crump, 2011; Fortunato et al., 2012). However, specific community compositions associated with specific land cover patterns or nutrient concentrations remains poorly explored in riverine communities, particularly in the water column.

At the local scale, biotic interactions have been reported to be primary drivers of variation in bacterial community structure (Fortunato and Crump, 2011), and this result is similar to that reported here in which associations were primarily observed among bacterial orders rather than nutrient and chemical concentrations by LSA. The finding that the majority of significant associations were observed among bacterial orders in the Mississippi River is also similar to observations of microbial communities in the English Channel (Gilbert et al., 2012), although the scale of these two ecosystems differ considerably in ecosystem type, study area, and nutritional availability. The local associations identified here may be particularly important when assessing community relationships in a lotic ecosystem prone to continual changes in nutrient availability and bacterial taxa as well as variation in residence time associated with seasonal variation and, potentially, land cover. A recent study of microbial communities in river sediments found that while primarily environmental drivers shaped community structure (Gibbons et al., 2014), dispersal dynamics and stochastic forces may play a minor role. When residence time is short, as might be expected in the late Spring and early Summer in this system, biotic interactions may play a stronger role in defining community dynamics than during later months when residence time increases. This possibility, as well as potential time-delayed effects on community structure in this ecosystem will require further study.

Despite the highly intercorrelated relationships among orders and seeming independence from local environmental and chemical parameters in the association network, significant directional relationships were inferred between both water chemistry, especially organic carbon and TDS concentrations, and order abundances as well as among bacterial orders alone at the regional scale via Bayesian analysis. These results suggest that physicochemical and nutrient concentrations may influence only a small number of taxa locally, but these effects may be propagated throughout the entire community, potentially as a result of interactions among taxa throughout this ecosystem. For example, the negative Bayesian correlation of temperature with *E. coli* concentration may indicate increases in abundance of other community members that out-compete this species, and *E. coli* is known to be a minority member of the community (Byappanahalli and Fujioka, 2004). Elucidation of these potential interactive effects have been previously utilized to create a predictive artificial neural network for microbial communities (Larsen et al., 2012). However, development of such a network was beyond the scope of the current study as few time points were analyzed.

Discrepancies between associations observed from local vs. regional analyses suggest that the spatial scale at which data are analyzed affects the conclusions drawn (Ruan et al., 2006; Xia et al., 2011; Gilbert et al., 2012). Future work will be required to validate whether the associations observed in this study represent actual community-level bacterial interactions, as this could not be determined here. A previous study indicated that responses to variation in nutrient concentration affect the distribution of functional traits in a community while taxonomic composition is habitat-specific (Comte and del Giorgio, 2009). Similarly, a study of geographically disparate freshwater cyanobacterial blooms found that functional traits were conserved among ecosystems despite variation in taxonomic community composition (Steffen et al., 2012). Based on these previous findings, community composition here may not reflect community adaptation to changes in nutrient concentrations but may instead be indicative of the bacteria contributed from natural runoff and anthropogenic impacts.

Furthermore, this study is restricted by the removal of aggregate bacteria, which have been shown to be phylogenetically and functionally diverse (Grossart, 2010), as well as the limitation to only bacterial orders that are represented in taxonomic classification databases. Complex trophic interactions are known to influence the bacterial community (Verreydt et al., 2012), and the lack of these associations between domains here may result in an over-simplified model of inter-specific associations among the total community. Finally, assessing bacterial associations when taxa are classified to orders may also reduce model complexity as bacterial orders can show significant diversity in functional traits, functional overlap, and differential survival ability. However, based on the shortness of sequence reads, more specific classification was not performed as taxonomic assignment to more specific levels have been shown to have poor accuracy in classification (Mizrahi-Man et al., 2013).

We hypothesized here that variation in major land coverage types would result in consistent directional shifts in the relative abundance of bacterial orders due to alteration of nutrient and chemical concentrations, as well as inputs of source-specific bacterial groups. Alteration of nutrient concentrations as a result of land coverage has been previously reported (Fisher et al., 2000; Miller et al., 2011), and these reports are generally consistent with the results presented here. Similarly, water quality has also been shown to be influenced by land cover in a predominantly urban setting (Tu, 2011). A previous study of stream sediments has further reported that variation in bacterial communities was significantly associated with impervious land cover associated with urbanization, although taxonomic assignments of variable OTUs was not addressed (Wang et al., 2011). Community variation characterized by shifts in abundances of specific taxa have also been observed in near-atmosphere air samples collected from forested, suburban, and agricultural areas (Bowers et al., 2011). Interestingly, the dominant groups identified in air samples—*Burkholderiales* and *Actinobacteria*—were also the most abundant groups identified in this study and were not correlated with any other abiotic parameter with the exception of a negative correlation between *Burkholderiales* abundance and cumulative (three-day) antecedent rainfall. The lack of correlations of these orders may result from their general ubiquity and high relative abundance, although it is important to note that the majority of variation is seen amongst taxa present in only moderate or minor abundances.

Although bacterial community structure could not be significantly associated with major land coverage by local analyses, abundances of many orders were correlated with land coverage when analyzed in the context of the entire dataset. Results of DFA suggested that only a few orders were significant in explaining variation in community structure based on patterns of land coverage, and several of these may be more useful targets to identify major non-point sources of contamination to the river or to identify biotic interactions influencing variation in community membership and/or structure. For example, increases in abundance of the orders *Aeromonadales* and *Nitrosomonadales* may serve as indicators for specifically urbanized contamination, while an increase in a specific order of *Gammaproteobacteria* may indicate the relative absence of anthropogenic impact. Lack of significant differences in abundance of many of these orders between developed and agricultural land could also imply that several groups are indicators of more generalized anthropogenic impacts (i.e., not specifically developed or agricultural, such as failure of septic systems), as has been previously suggested (Staley et al., 2013). Frequent matching of OTU sequences from orders associated with developed land cover to isolates from the wastewater treatment process suggest that these orders may be contributed as a result of effluent outfall. Similarly, matching of forest-associated OTUs with isolates from only freshwater bodies lends credit to the conclusion that these orders are reflective of more pristine, unimpacted conditions. It should be noted, however, that BLAST searching was far from exhaustive due to the size of the GenBank database, and isolates from many other sources may share identity with the OTU sequences queried. Use of computational algorithms such as SourceTracker (Knights et al., 2011) which employs an OTU-based approach characterizing source and sink communities will provide more objective determination of sources of OTUs, as demonstrated recently (Newton et al., 2013). However, use of these methods will require knowledge of specific sources and extensive characterization of their microbial communities, and this data was not collected in the current study.

Importantly, the concentration of *E. coli*, which is commonly used as an indicator of surface water quality and human health risk (United States Environmental Protection Agency, 2002, 2012), was not correlated with other measures of water quality (e.g., nitrogen concentration) or abundances of several orders that include human pathogens (e.g., *Enterobacteriales*) among the entire dataset. Furthermore, *E. coli* concentration was not significantly associated with the abundance of bacterial orders by local or Bayesian analyses, suggesting a poor relationship between this species and the overall bacterial community structure. *E. coli* concentrations measured here only twice exceeded the Environmental Protection Agency's one-time sampling threshold of 235 CFU 100 ml^−1^ (United States Environmental Protection Agency, 2002), suggesting that risk of human pathogens during the sampling period may have been limited, although the presence of pathogens or of fecal contamination during this study was not determined. However, previous studies have suggested that factors including nutrient concentrations, land coverage, and the surrounding bacterial community are all potential factors associated with pathogen presence and activity (Viau et al., 2011; Williams et al., 2012), therefore *E. coli* may be an even poorer indicator of water quality than previously thought.

This study highlights the complexity of factors that influence bacterial community structure locally and regionally in a complex riverine ecosystem. Among all analyses, organic carbon and TDS were observed to be among the primary environmental factors influencing both diversity and the abundance of specific bacterial orders. These parameters were also regionally associated with specific land cover types suggesting that specific anthropogenic impacts alter the chemistry of this riverine ecosystem and contribute non-indigenous bacteria resulting in shifts in the overall bacterial community. Furthermore, this study is among the first to suggest that specific bacterial orders in the water column may be indicative of specific types of non-point source contamination, and may serve as more informative indicators of ecosystem impairment than traditional indicator bacteria. Further interrogation of the associations and networks proposed here will better allow regulatory agencies and resource managers to determine if contamination is a result of relatively local, potentially point sources, or may be due to an accumulation of chemicals from more diffuse, point and non-point sources upstream.

### Conflict of interest statement

The authors declare that the research was conducted in the absence of any commercial or financial relationships that could be construed as a potential conflict of interest.

## References

[B1] Aronesty E. (2013). Comparison of sequencing utility programs. Open Bioinforma. J. 7, 1–8 10.2174/1875036201307010001

[B2] Balogh S. J. Engstrom D. R. Almendinger J. E. McDermott C. Hu J. Nollet Y. H. (2009). A sediment record of trace metal loadings in the Upper Mississippi River. J. Paleolimnol. 41, 623–639 10.1007/s10933-008-9295-2

[B3] Balogh S. J. Engstrom D. R. Almendinger J. E. Meyer M. L. Johnson D. K. (1999). History of mercury loading in the Upper Mississippi River reconstructed from the sediments of Lake Pepin. Environ. Sci. Technol. 33, 3297–3302 10.1021/es9903328

[B4] Bowers R. M. McLetchie S. Knight R. Fierer N. (2011). Spatial variability in airborne bacterial communities across land-use types and their relationship to the bacterial communities of potential source environments. ISME J. 5, 601–612 10.1038/ismej.2010.167 21048802 PMC3105744

[B5] Brandsma J. Martinez J. M. Slagter H. A. Evans C. Brussaard C. P. D. (2013). Microbial biogeography of the North Sea during summer. Biogeochemistry 113, 119–136 10.1007/s10533-012-9783-3

[B6] Bray J. R. Curtis J. T. (1957). An ordination of the upland forest communities of southern Wisconsin. Ecol. Monogr. 27, 325–349 10.2307/1942268

[B7] Byappanahalli M. Fujioka R. (2004). Indigenous soil bacteria and low moisture may limit but allow faecal bacteria to multiply and become a minor population in tropical soils. Water Sci. Technol. 50, 27–32 15318482

[B8] Caporaso J. G. Lauber C. L. Walters W. A. Berg-Lyons D. Huntley J. Fierer N. (2012). Ultra-high-throughput microbial community analysis on the Illumina HiSeq and MiSeq platforms. ISME J. 6, 1621–1624 10.1038/ismej.2012.8 22402401 PMC3400413

[B9] Clarke K. R. (1993). Non-parametric multivariate analyses of changes in community structure. Aust. J. Ecol. 18, 117–143 10.1111/j.1442-9993.1993.tb00438.x19830478

[B10] Cole J. R. Wang Q. Cardenas E. Fish J. Chai B. Farris R. J. (2009). The Ribosomal Database Project: improved alignments and new tools for rRNA analysis. Nucleic Acids Res. 37, D141–D145 10.1093/nar/gkn879 19004872 PMC2686447

[B11] Comte J. del Giorgio P. A. (2009). Links between resources, C metabolism and the major components of bacterioplankton community structure across a range of freshwater ecosystems. Environ. Microbiol. 11, 1704–1716 10.1111/j.1462-2920.2009.01897.x 19508562

[B12] Drury B. Rosi-Marshall E. Kelly J. J. (2013). Wastewater treatment effluent reduces the abundance and diversity of benthic bacterial communities in urban and suburban rivers. Appl. Environ. Microbiol. 79, 1897–1905 10.1128/AEM.03527-12 23315724 PMC3592216

[B13] Edgar R. C. Haas B. J. Clemente J. C. Quince C. Knight R. (2011). UCHIME improves sensitivity and speed of chimera detection. Bioinformatics 27, 2194–2200 10.1093/bioinformatics/btr381 21700674 PMC3150044

[B14] Ellis J. B. (2006). Pharmaceutical and personal care products (PPCPs) in urban receiving waters. Environ. Pollut. 144, 184–189 10.1016/j.envpol.2005.12.018 16500738

[B15] Excoffier L. Smouse P. E. Quattro J. M. (1992). Analysis of molecular variance inferred from metric distances among DNA haplotypes—application to human mitochondrial DNA restriction data. Genetics 131, 479–491 1644282 10.1093/genetics/131.2.479 PMC1205020

[B16] Fisher D. Steiner J. Endale D. Stuedemann J. Schomberg H. Franzluebbers A. (2000). The relationship of land use practices to surface water quality in the Upper Oconee Watershed of Georgia. For. Ecol. Manage. 128, 39–48 10.1016/S0378-1127(99)00270-4

[B17] Fortunato C. S. Crump B. C. (2011). Bacterioplankton community variation across river to ocean environmental gradients. Microb. Ecol. 62, 374–382 10.1007/s00248-011-9805-z 21286702

[B18] Fortunato C. S. Herfort L. Zuber P. Baptista A. M. Crump B. C. (2012). Spatial variability overwhelms seasonal patterns in bacterioplankton communities across a river to ocean gradient. ISME J. 6, 554–563 10.1038/ismej.2011.135 22011718 PMC3280145

[B19] Fry J. Xian G. Jin S. Dewitz J. Homer C. Yang L. (2011). Completion of the 2006 national land cover database for the conterminous United States. Photogramm. Eng. Remote Sens. 77, 858–864

[B20] Gibbons S. M. Jones E. Bearquiver A. Blackwolf F. Roundstone W. Scott N. (2014). Human and environmental impacts on river sediment microbial communities. PLoS ONE 9:e97435 10.1371/journal.pone.0097435 24841417 PMC4026135

[B21] Gilbert J. A. Field D. Swift P. Newbold L. Oliver A. Smyth T. (2009). The seasonal structure of microbial communities in the Western English Channel. Environ. Microbiol. 11, 3132–3139 10.1111/j.1462-2920.2009.02017.x 19659500

[B22] Gilbert J. A. Steele J. A. Caporaso J. G. Steinbrueck L. Reeder J. Temperton B. (2012). Defining seasonal marine microbial community dynamics. ISME J. 6, 298–308 10.1038/ismej.2011.107 21850055 PMC3260500

[B23] Grossart H. P. (2010). Ecological consequences of bacterioplankton lifestyles: changes in concepts are needed. Environ. Microbiol. Rep. 2, 706–714 10.1111/j.1758-2229.2010.00179.x 23766274

[B24] Knights D. Kuczynski J. Charlson E. S. Zaneveld J. Mozer M. C. Collman R. G. (2011). Bayesian community-wide culture-independent microbial source tracking. Nat. Methods 8, 761–U107 10.1038/nmeth.1650 21765408 PMC3791591

[B25] Larsen P. E. Field D. Gilbert J. A. (2012). Predicting bacterial community assemblages using an artificial neural network approach. Nat. Methods 9, 621–625 10.1038/nmeth.1975 22504588

[B26] Lozupone C. Knight R. (2005). UniFrac: a new phylogenetic method for comparing microbial communities. Appl. Environ. Microbiol. 71, 8228–8235 10.1128/AEM.71.12.8228-8235.2005 16332807 PMC1317376

[B27] Miller J. D. Schoonover J. E. Williard K. W. J. Hwang C. R. (2011). Whole catchment land cover effects on water quality in the lower Kaskaskia River watershed. Water Air Soil Pollut. 221, 337–350 10.1007/s11270-011-0794-9

[B28] Mizrahi-Man O. Davenport E. R. Gilad Y. (2013). Taxonomic classification of bacterial 16S rRNA genes using short sequencing reads: evaluation of effective study designs. PLoS ONE 8:e53608 10.1371/journal.pone.0053608 23308262 PMC3538547

[B29] Newton R. J. Bootsma M. J. Morrison H. G. Sogin M. L. McLellan S. L. (2013). A microbial signature approach to identify fecal pollution in the waters off an urbanized coast of Lake Michigan. Microb. Ecol. 65, 1011–1023 10.1007/s00248-013-0200-9 23475306 PMC4084971

[B30] Pereira W. E. Hostettler F. D. (1993). Nonpoint-source contamination of the Mississippi River and its tributaries by herbicides. Environ. Sci. Technol. 27, 1542–1552 10.1021/es00045a008

[B31] Portillo M. C. Anderson S. P. Fierer N. (2012). Temporal variability in the diversity and composition of stream bacterioplankton communities. Environ. Microbiol. 14, 2417–2428 10.1111/j.1462-2920.2012.02785.x 22626459

[B32] Pruesse E. Quast C. Knittel K. Fuchs B. M. Ludwig W. G. Peplies J. (2007). SILVA: a comprehensive online resource for quality checked and aligned ribosomal RNA sequence data compatible with ARB. Nucleic Acids Res. 35, 7188–7196 10.1093/nar/gkm864 17947321 PMC2175337

[B33] Ruan Q. Dutta D. Schwalbach M. S. Steele J. A. Fuhrman J. A. Sun F. (2006). Local similarity analysis reveals unique associations among marine bacterioplankton species and environmental factors. Bioinformatics 22, 2532–2538 10.1093/bioinformatics/btl417 16882654

[B34] Russell T. A. Weller L. (2012). State of the River Report: Water Quality and River Health in the Metro Mississippi River. Saint Paul, MN. Available online at: http://stateoftheriver.com/state-of-the-river-report/

[B35] Schilling K. E. Chan K. S. Liu H. Zhang Y. K. (2010). Quantifying the effect of land use land cover change on increasing discharge in the Upper Mississippi River. J. Hydrol. 387, 343–345 10.1016/j.jhydrol.2010.04.019

[B36] Schloss P. D. Westcott S. L. Ryabin T. Hall J. R. Hartmann M. Hollister E. B. (2009). Introducing mothur: open-source, platform-independent, community-supported software for describing and comparing microbial communities. Appl. Environ. Microbiol. 75, 7537–7541 10.1128/AEM.01541-09 19801464 PMC2786419

[B37] Shannon P. Markiel A. Ozier O. Baliga N. S. Wang J. T. Ramage D. (2003). Cytoscape: a software environment for integrated models of biomolecular interaction networks. Genome Res. 13, 2498–2504 10.1101/gr.1239303 14597658 PMC403769

[B38] Smith V. A. Yu J. Smulders T. V. Hartemink A. J. Jarvis E. D. (2006). Computational Inference of Neural Information Flow Networks. PLoS Comput. Biol. 2:e161 10.1371/journal.pcbi.0020161 17121460 PMC1664702

[B39] Sogin M. L. Morrison H. G. Huber J. A. Mark Welch D. Huse S. M. Neal P. R. (2006). Microbial diversity in the deep sea and the underexplored “rare biosphere.” Proc. Natl. Acad. Sci. U.S.A. 103, 12115–12120 10.1073/pnas.0605127103 16880384 PMC1524930

[B40] Staley C. Unno T. Gould T. J. Jarvis B. Phillips J. Cotner J. B. (2013). Application of Illumina next-generation sequencing to characterize the bacterial community of the Upper Mississippi River. J. Appl. Microbiol. 115, 1147–1158 10.1111/jam.12323 23924231

[B41] Steffen M. M. Li Z. Effler T. C. Hauser L. J. Boyer G. L. Wilhelm S. W. (2012). Comparative metagenomics of toxic freshwater cyanobacteria bloom communities on two continents. PLoS ONE 7:e44002 10.1371/journal.pone.0044002 22952848 PMC3430607

[B42] Tu J. (2011). Spatial and temporal relationships between water quality and land use in northern Georgia, USA. J. Integr. Environ. Sci. 8, 151–170 10.1080/1943815X.2011.577076

[B43] United States Environmental Protection Agency (2002). EPA-823-B-02-003. Implementation Guidance for Ambient Water Quality Criteria for Bacteria.

[B44] United States Environmental Protection Agency (2007). Science Advisory Board (SAB) Hypoxia Panel Draft Advisory Report. 2013. Available online at: http://www.epa.gov/sab/pdf/11-19-07_hap_draft.pdf

[B45] United States Environmental Protection Agency (2012). Recreational Water Quality Criteria. 820-F-12-058. United States Environ. Prot. Agency Off. Water.

[B46] Unno T. Di D. Y. Jang J. Suh Y. S. Sadowsky M. J. Hur H. G. (2012). Integrated online system for a pyrosequencing-based microbial source tracking method that targets *Bacteroidetes* 16S rDNA. Environ. Sci. Technol. 46, 93–98 10.1021/es201380c 21780740

[B47] Unno T. Jang J. Han D. Kim J. H. Sadowsky M. J. Kim O. S. (2010). Use of barcoded pyrosequencing and shared OTUs to determine sources of fecal bacteria in watersheds. Environ. Sci. Technol. 44, 7777–7782 10.1021/es101500z 20853824

[B48] Verreydt D. De Meester L. Decaestecker E. Villena M.-J. Van Der Gucht K. Vannormelingen P. (2012). Dispersal-mediated trophic interactions can generate apparent patterns of dispersal limitation in aquatic metacommunities. Ecol. Lett. 15, 218–226 10.1111/j.1461-0248.2011.01728.x 22221744

[B49] Viau E. J. Goodwin K. D. Yamahara K. M. Layton B. A. Sassoubre L. M. Burns S. L. (2011). Bacterial pathogens in Hawaiian coastal streams - associations with fecal indicators, land cover, and water quality. Water Res. 45, 3279–3290 10.1016/j.watres.2011.03.033 21492899

[B50] Wang S.-Y. Sudduth E. B. Wallenstein M. D. Wright J. P. Bernhardt E. S. (2011). Watershed urbanization alters the composition and function of stream bacterial communities. PLoS ONE 6:e22972 10.1371/journal.pone.0022972 21857975 PMC3155513

[B51] Wiener J. G. Sandheinrich M. B. (2010). Contaminants in the Upper Mississippi River: historic trends, responses to regulatory controls, and emerging concerns. Hydrobiologia 640, 49–70 10.1007/s10750-009-0064-7

[B52] Williams A. P. Quilliam R. S. Thorn C. E. Cooper D. Reynolds B. Jones D. L. (2012). Influence of land use and nutrient flux on metabolic activity of *E. coli* O157 in river water. Water Air Soil Pollut. 223, 3077–3083 10.1007/s11270-012-1090-z

[B53] Xia L. C. Steele J. A. Cram J. A. Cardon Z. G. Simmons S. L. Vallino J. J. (2011). Extended local similarity analysis (eLSA) of microbial community and other time series data with replicates. BMC Syst. Biol. 5 Suppl. 2, S15 10.1186/1752-0509-5-S2-S15 22784572 PMC3287481

[B54] Zhang S. Y. Zhang Q. A. Darisaw S. Ehie O. Wang G. D. (2007). Simultaneous quantification of polycyclic aromatic hydrocarbons (PAHs), polychlorinated biphenyls (PCBs), and pharmaceuticals and personal care products (PPCPs) in Mississippi River water, in New Orleans, Louisiana, USA. Chemosphere 66, 1057–1069 10.1016/j.chemosphere.2006.06.067 16884762

