# Supplementary material for: Bacterial community structure is indicative of chemical inputs in the Upper Mississippi River
Source: Front Microbiol. 2014 Oct 8;5:524. doi: 10.3389/fmicb.2014.00524 (PMC4189419; doi:10.3389/fmicb.2014.00524)
Supplement: Supplementary file 1 [file DataSheet1.DOCX]

***Supplementary Material***

**Bacterial Community Structure is Indicative of Specific Chemical Inputs in the Upper Mississippi River**

**Christopher Staley^1^, Trevor J. Gould^1,2^, Ping Wang^1^, Jane Phillips^2^, James B. Cotner^3^, and Michael J. Sadowsky^1,4,*^**

^1^BioTechnology Institute,

^2^Biology Program,

^3^Department of Ecology, Evolution and Behavior,

^4^Department of Soil, Water and Climate, University of Minnesota, St. Paul, MN

^*^Corresponding Author: Michael J. Sadowsky, BioTechnology Institute, University of Minnesota, 140 Gortner Lab, 1479 Gortner Ave, Saint Paul, MN 55108; Phone: (612)-624-2706, Email: [sadowsky@umn.edu](mailto:sadowsky@umn.edu)

**Supplementary Figures**

**Figure S1.** Primary network analysis of well-correlated, significant, local similarity relationships (*P* < 0.05, Q < 0.003, -0.7 < Spearman’s r < 0.7) among bacterial orders (blue circles), physicochemical parameters (green diamonds), chemicals (nutrients and ions; yellow hexagons), and land coverage (orange squares). Black edges indicate positive local similarity scores and dashed, red edges are negative, edge length is arbitrary. Spearman’s r values are shown on the edges, except when orders were only detected in one sample and could not be analyzed for correlation. Numbers in parentheses indicate group (*Acidobacteria*), family (*Cyanobacteria*), or subdivision (*Verrucomicrobia*) and asterisks (*) indicate orders unclassified or designated *incertae sedis*.

**FigureS2.** Smaller networks of well-correlated, significant local similarity relationships (*P* < 0.05, Q < 0.003, -0.7 < Spearman’s r < 0.7) among bacterial orders (blue circles) and xenobiotic compounds (red squares). Black edges indicate positive local similarity scores and edge length is arbitrary. Spearman’s r values are shown on the edges. Numbers in parentheses indicate group (*Acidobacteria*) or family (*Cyanobacteria*).

**Figure S3.** Correlation coefficients (r) relating relative abundance of orders to total developed or total forested area within HUC boundaries. Total developed and forested land area were negatively correlated (r = -0.581, *P* < 0.001). Superscripts indicate significant positive correlations while subscripts indicate negative correlations (α = 0.05). Correlations are shown for *E. coli* (*), carbon (†), nitrate/nitrite (‡), orthophosphate (§), and TDS (¶). Non-italic names indicate orders *incertae sedis.*

**Figure S4.** Correlation coefficients (r) relating relative abundance of orders to pasture or total forested area within HUC boundaries. Superscripts indicate significant positive correlations while subscripts indicate negative correlations (α = 0.05). Correlations are shown for *E. coli* (*), carbon (†), nitrate/nitrite (‡), orthophosphate (§), and TDS (¶). Non-italic names indicate orders *incertae sedis.*
