# Supplementary figures and images for: Bacterial community structure is indicative of chemical inputs in the Upper Mississippi River
Source: Front Microbiol. 2014 Oct 8;5:524. doi: 10.3389/fmicb.2014.00524 (PMC4189419; doi:10.3389/fmicb.2014.00524)

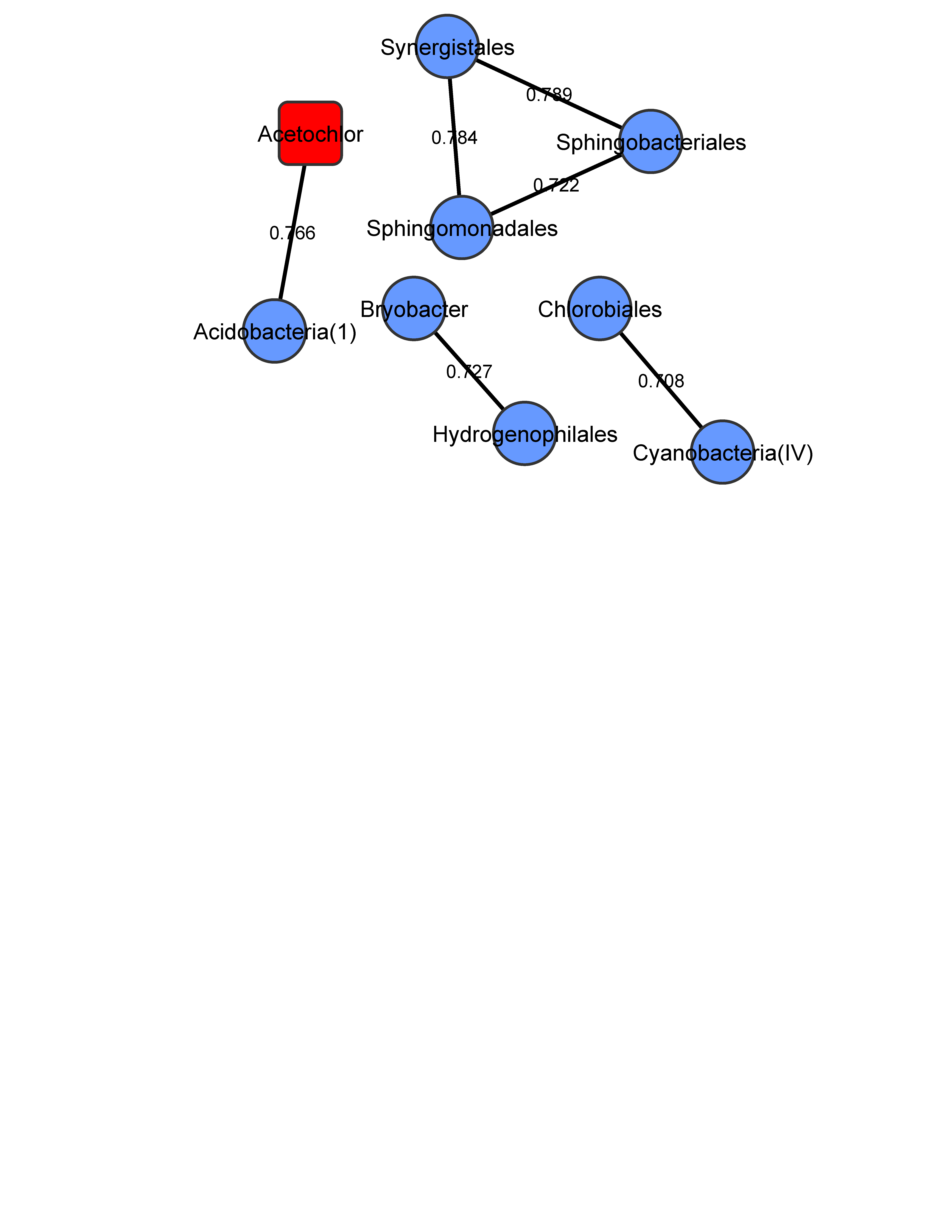

Supplement: Supplementary file 3 [file Image2.TIFF]
